# Cutaneous melanoma: cost of illness under Brazilian health system perspectives

**DOI:** 10.1186/s12913-021-06246-1

**Published:** 2021-03-29

**Authors:** Cassia Rita Pereira da Veiga, Claudimar Pereira da Veiga, Alceu Souza, Alberto Julius Alves Wainstein, Andreia Cristina de Melo, Ana Paula Drummond-Lage

**Affiliations:** 1 Departamento de Administração Geral e Aplicada (DAGA), Escola de Administração, Universidade Federal do Paraná (UFPR), Lothário Meissner 632, Jardim Botânico, Curitiba, PR 80210-170 Brazil; 2 Pontifícia Universidade Católica do Paraná (PUCPR), Imaculada Conceição 1155, Curitiba, PR 80215-901 Brazil; 3 Faculdade de Ciências Médicas de Minas Gerais (FCMMG), Alameda Ezequiel Dias 275, Belo Horizonte, MG 30130-110 Brazil; 4 Instituto Nacional de Câncer (INCA), Rio de Janeiro, Praça Cruz Vermelha 23, Rio de Janeiro, RJ 20230-130 Brazil

**Keywords:** Brazilian health care system, Cutaneous melanoma, Cost-of-illness, Executional cost management, Structural cost management

## Abstract

**Background:**

The landscape of cutaneous melanoma (CM) diagnosis, staging, prognosis, and treatment has undergone fundamental changes in the past decade. While the benefits of new health resources are recognized, there is a distinct lack of accurate cost-of-illness information to aid healthcare decision makers.

**Methods:**

The cost-of-illness study for CM was conducted from the perspective of two health systems in Brazil: the public health system (Unified Health System, SUS) and the private health system (Health Management Organization, HMO). The study considered the direct medical cost in a bottom-up analysis, using melanoma incidence, knowledge of the disease’s progression, and the overall survival rates. The executional costs for the complete healthcare delivery cycle were investigated considering different disease stages and possible clinical course variations. The structural cost was assessed qualitatively considering the health value chain in Brazil.

**Results:**

CM represents a critical financial burden in Brazil, and the cost of illness varied according to the health system and by stage at diagnosis. HMO patient costs are approximately 10-fold and 90-fold more than a SUS patient in the early-stage and advanced disease, respectively. Overall, spending on advanced disease patients can be up to 34-fold (SUS) or 270-fold (HMO) higher than that required for the early-stage disease. Given the massive amount of resources spent by the SUS and HMO, significant efforts must be made to improve the health value chain to deliver the right mix of medical care goods and services using available resources.

**Conclusion:**

The cost-of-illness study for CM has the potential to inform policymakers and decision-makers regarding the economic burden that melanoma impose on a society in terms of the use of health care services, assisting them in making projections of future health care costs and resource allocation decisions. We believe that cost-of-illness analysis from a strategic perspective could be of help in assessing executional costs and be used to support the change in structural costs required for long-term strategies related to the health value chain.

**Supplementary Information:**

The online version contains supplementary material available at 10.1186/s12913-021-06246-1.

## Background

The landscape of cutaneous melanoma (CM) diagnosis, staging, prognosis, and treatment has been fundamentally altered in the past decade. A historical review shows a change in the size of resection margins, the introduction of the sentinel lymph node biopsy, and, since 2011, the approval of new and more effective systemic treatments. Furthermore, the development and dissemination of new imaging techniques, such as dermoscopy, ultrasound, magnetic resonance, and positron emission tomography have led to a more accurate diagnosis and staging of patients [1, 2].

Currently, the definition of the diagnostic and therapeutic strategy for melanoma patients involves choosing between multiple alternative courses of action that impact cost structure and performance for patient survival. On the other hand, for most healthcare systems, the unaffordability of new technologies is a widespread phenomenon [3], and a large percentage of patients have restricted access to the necessary healthcare resources [4, 5]. In particular, Brazil has a population of over 200 million, and almost 76% of the population have to rely exclusively on public healthcare, which is very limited regarding access to health innovations and medical aid [6, 7]. Moreover, the Brazilian incidence rate of melanoma has doubled since the year 2000, and should increase in coming years because of an aging population trend [7], which will result in a heavier CM financial burden.

The issue of healthcare costs has become increasingly important over the years. In the literature, there has been growing interest on the relative cost-effectiveness and sustainability of delivering high-quality cancer care, with most emphasis given to cost control of new technologies [8, 9]. While the benefits of these innovations are recognized, there is a distinct lack of accurate cost information for healthcare decisions makers [10]. Most accounting studies are conducted outside the scope of healthcare research. They address issues mainly related to comparing the cost of multiple alternative courses.

In this study, we examine the issue from the broader perspective of Shank and Vijay [11], and Anderson and Dekker [12, 13] that strategic cost management (SCM) is composed of executional cost management and structural cost management and both can be used in cost-of-illness studies. It is possible to define executional cost management as the cost management of resources used to deliver a health care cycle. In turn, structural cost management refers to cost management based on the health value chain, considering budget limitations, inequity in access to care, and resource affordability [11–13]. Although managers continue to pursue efficiency and effectiveness in individual processes, significant improvements are obtained by structural cost management across the value chain. In this respect, we used SCM concepts to evaluate cost performance and provide the necessary knowledge to identify interventions that can reduce the melanoma burden from a strategic perspective [11]. In other words, the purpose of this cost-of-illness study was to evaluate and correlate the healthcare resources used for CM diagnosis and treatment with: (i) short-term tactics through cost driver analysis (executional cost management), and (ii) long-term strategy through the re-engineering of the value chain that was compatible with different cost structures (structural cost management).

Although there is no national database with historical series of the consumption of health resources per patient in Brazil, cost-of-illness studies in Brazil are feasible because the general costing information is available and publicly accessible in health information systems. Cost-of-illness studies allow an estimation of the global melanoma cost from the perspective of different health systems in Brazil. Although their relevance has been questioned, cost-of-illness studies can provide strategic information for decision making on the allocation of health resources [14].

The current work largely contributes to the literature by: (i) developing a disease model that simulates the reality of the CM patient’s journey and considers the performance of the health resources, that is, this work evaluates both costs and outcomes, which represented a significant limitation of previous cost-of-illness studies [15]; (ii) validating the new pattern of use of health resources to diagnose and treat melanoma in Brazilian health systems, as many changes have occurred in the CM scenario after previous publications related to the theme in Brazil [16]; (iii) evaluating cost of illness for melanoma with analysis of both executional and structural cost management to complete the health care delivery cycle of a CM patient’s journey, unlike most Brazilian oncology studies, which focus on cost-effectiveness analysis of interventions used in advanced disease [9, 17, 18]; (iv) achieving progress for the cost-of-illness literature applied to oncology, thereby gaining strategic insights to improve the efficiency of the health value chain for cancer, which despite its importance, is a theme unexplored in Brazil. This study is the first to evaluate cost of illness for melanoma using a cost mapping tool with strategic analysis from the SCM perspective.

## Methods

Cost-of-illness studies are largely driven by data availability and the choice of methodology can influence the magnitude of the estimates [14]. In the absence of a national database with a historical series of health resource consumption per patient in Brazil, this research is composed of three steps. First, we proposed a disease model inspired by Markov models [19] using secondary data supported by the melanoma literature. The disease’s total financial burden depends on the natural history of the disease and of the health resources used since the initial diagnosis. As cost-of-illness studies attempt to quantify the magnitude of an association between disease and cost, the disease model needed to ensure knowledge of the disease’s progression and the overall survival rates at each stage of the CM patient in the diagnosis [14, 20].

Second, based on the executional cost management approach [13], we evaluated both the main cost drivers and the total cost to complete the health care delivery cycle for the first 3 years after diagnosis. This research was limited to 3 years because overall survival data were not available in the extended follow-up of clinical trials at the time of this study. We used a cost mapping tool in a previously published database [21]. Finally, we discussed the results based on the cost drivers in accordance with the structural cost management approach in the last step [11, 12, 22]. The objective was to correlate cost drivers with potential changes in the healthcare value chain in Brazil. In this stage, we used qualitative information from publications about Brazilian health systems that showed the main barriers to improving efficiency and reducing costs in the health value chain.

The cost-of-illness study for melanoma was conducted from the perspective of two health systems in Brazil: the public health system (Unified Health System, SUS) and the private health system (Health Management Organization, HMO). The SUS represents a significant step forward that guarantees universal healthcare for all Brazilians, although regular access to essential medical care remains a distant ideal. Currently, 76% of the population depend exclusively on the SUS [23]. In the public health system, a fixed amount of resources is allocated for patients with a specific diagnosis, and decision making in healthcare reflects a restriction imposed by the lack of financing.

On the other hand, there are more than 700 different HMOs in the Brazilian supplementary healthcare system, and almost 70% of private health plans are paid for by companies to benefit their employees [23]. The National Supplementary Healthcare Agency (ANS) regulates the HMOs in their relations with healthcare service providers and consumers. Due to the incorporation of high-cost procedures in recent years, supplementary healthcare in Brazil has suffered a loss of efficiency and sustainability.

**Fig. 1:**
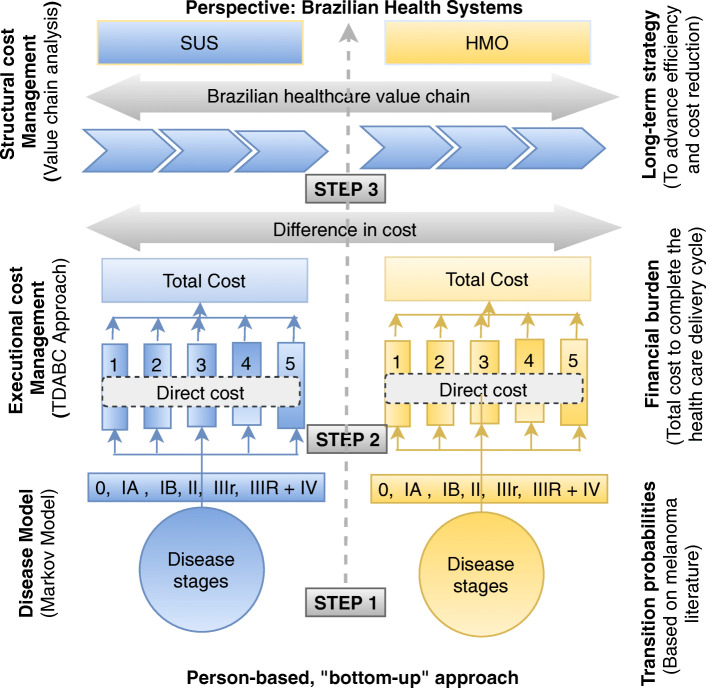
Steps of the cost-of-illness research methodology to CM patients in the Brazilian Health care Systems (adapted from Larg and Moss, 2011) [14]. SUS = Public health system (Unified Health System); HMO = Private health system (Health Management Organization); TDABC = Time-driven activity-based costing; CM = Cutaneous melanoma

The cost-of-illness study for melanoma considered the direct medical cost in a bottom-up analysis, which consists of estimating of the types of health resources consumed by an individual throughout their journey with the disease, multiplied by the respective unit costs [14]. Direct non-medical and indirect costs were not included in the study because it was not possible to quantify them by clinical trial data and because of the absence of standardized and reliable data in Brazil. The assessment of intangible costs is not applicable to the perspective used in the study. Figure 1 illustrates and details the three steps of the methodology used in this research.

### Disease model and transition probabilities

**Fig. 2:**
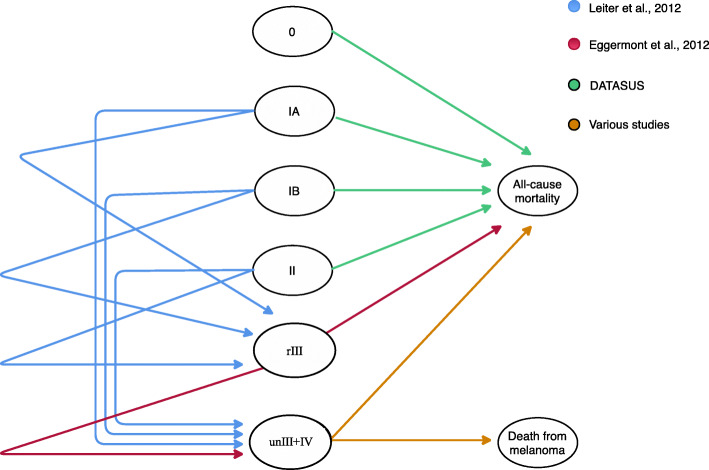
Disease model and the transition probabilities (adapted from Tromme et al., 2016) [19]

Figure 2 shows the disease model and the general transition probabilities. Tromme et al. [19] was chosen as the base model because they compared the disease burden to different melanoma stages at the localized, node, and metastatic stages. This division of the melanoma burden may help to establish priorities for healthcare resource allocations. Moreover, this reference shows ways to evaluate both costs and outcomes of a CM patient’s journey. Adaptations of the disease model and the general transition probabilities are detailed in this subsection. CM patients could start at one of the six diagnosis stages, 0, IA, IB, II, rIII (resectable disease) or unIII + IV (stage III unresectable + metastatic disease). They could either stay at the same stage or change to another stage depending on the disease model and transition probabilities. We analyzed the first 3 years after the diagnosis that was considered when the patients were 60 years of age [7]. They could remain in the model until death by malignant tumor or achieve life expectancy [24]. The transition probabilities of recurrences from stages IA, IB, or II to stage rIII were based on the recurrence-free survival rate presented in Leiter et al. [25]. Of all the initial recurrences, 77% were considered locoregional or affected the regional lymph nodes, and 23.0% were classified as distant metastases [25].

Since there is no consensus on the adjuvant treatment option in CM patients [21], this study considered negative pathological results for the sentinel lymph node, a situation in which adjuvant treatment is not recommended. Anti-PD-1 treatments were not considered in the disease model’s adjuvant treatment options because to date there is no available information on overall survival [26, 27]. The transition probabilities from stage rIII to recurrence to stage unIII+IV or to death were derived from the annual rate of recurrence-free survival and the annual rate of overall survival presented in the observation arm of Eggermont et al. [28].

The probabilities of transition from the first line to subsequent stage IV treatment lines were based on the systemic treatment of choice based on previously published Brazilian research [21]. They were derived directly from estimated annual rates of both overall survival and progression-free survival at years 1 to 3. The first-line treatment options considered were chemotherapy [29, 30], anti-PD-1 treatment (alone or in combination with anti-CTLA-4 treatment) [31] or, the use of combined BRAF and MEK inhibition [32]. The model considered that patients who had a recurrence of the disease moved to the second and subsequent line of therapy: chemotherapy [33], anti-PD-1 treatment [33], anti-CTLA-4 treatment [34], combined BRAF and MEK inhibition [35], or palliative care [36].

At Stage IV, the transition probabilities model classified HMO patients based on BRAF status and disease volume, since Brazilian oncologists choose different therapeutic options according to these parameters [21]. The transition probabilities model considered patients with BRAF V600-mutated melanoma and a high (MBHV) or low (MBLV) volume of disease, as well as BRAF wild-type patients with a high (WBHV) or low (WBLV) volume of disease. SUS patients with an advanced disease did not receive these classifications because the systemic treatment involved only chemotherapy in the first line of treatment [21]. Further information used to develop the disease model and transition probabilities, such as the disease’s progression rate and the overall survival rate, can be found in the supplementary information.

Although the model aimed to simulate reality as much as possible, some simplifying assumptions had to be made: (1) transition probabilities were assumed to be the same for both genders and all ages over 60 years; (2) hazard rates for a second cutaneous melanoma were not considered, although previous studies have shown that CM patients may be at greater risk of subsequent CM or non-CM [37]; (3) at the time of this study, the most recent data available did not allow the subclassification of stages II and III, but new subcategorization of the staging of melanoma will continue to evolve to enable better care [38]; (4) the diagnosis of CM was made within 1 month for both HMO and SUS patients, but this time may be longer [39].

### Executional cost management: financial burden of complete health care delivery cycle

Brazilian health care is fragmented by facility or specialty, and this obstacle hinders accurate cost measurements. To overcome these challenges, we applied the time-drivenactivity-based costing (TDABC) approach [40], a feasible tool for comparing relative resource utilization that exploits time equations without increasing the model’s complexity. Another benefit of implementing a TDABC approach is the knowledge it generates regarding the resource utilization efficiencies employing process mapping. The TDABC addresses many executional cost management issues, allowing us to investigate the main cost drivers and the total cost for the complete healthcare delivery cycle using resource consumption time based on the disease model. The TDABC’s focus is on a functional level to verify whether accounting data can detect any economies for the healthcare system.

Although treating cancer is higher than the cost of treating other chronic medical conditions [41], only recently has the TDABC approach been applied to oncology healthcare [42]. In this study, the TDABC was applied to evaluate the financial burden of CM patients at different disease stages from diagnosis up to 3 years of follow up from the perspective of two Brazilian payers: the SUS and HMO. In addition to the total financial burden, the costs were also analyzed based on five groups of different medical resources: outpatient visits, laboratory exams, imaging exams, surgery and histopathological analysis, and drugs. Thus, the main cost drivers and the total cost structure for the complete healthcare delivery cycle to CM patients were investigated considering different disease stages and possible variations in their clinical course (based on the disease model).

The type and frequency of healthcare resources used in CM patients were evaluated in a previous Brazilian study [21], which is the only behavioral study applied to a Brazilian group of medical professionals who diagnose and treat melanoma. That study assessed the type and quantity of health resources used for the complete delivery cycle of health care at different stages of CM patient evolution in the Brazilian public and private health system. The TDABC approach applied to a database from published Brazilian research ensured that only costs related to the CM were attributed to total cost. Despite the limitations inherent to using primary data from previous research [21], the required strategic information would not be accessible otherwise.

The unit cost of each resource was obtained through a unified system of information management of procedures, medications, orthoses, prostheses, and special materials (SIGTAP) [43] for the SUS and by the Brazilian hierarchy classification of medical procedures (CBHPM^1^) [44–46] for HMO. The CBHPM allows up to 20% of the procedure cost, according to regionalization and free negotiation between the parties. The SIGTAP allows an increase of a total percentage of the procedure cost linked to a specific health care provider qualification (more than 10%). To simulate this cost variation, we used Oracle Crystal Ball Software.

The medical procedure codes most commonly applied to the CM patient considering all the pricing rules of the SIGTAP and CHBPM were chosen. Regarding cancer therapy costs, the HMO costs were estimated based on the drug list (ICMS tax 18%^2^) published by Ministers of the Drug Market Regulation Chamber (CMED) [47]. Cancer therapy costs in the SUS are controlled by the Authorization for High Complexity Procedures (APAC) system, integrating specific policies of the Ministry of Health. Each APAC has a particular sum for reimbursement, according to the type of cancer therapy used and treatment line. When applicable, this study considered a dose regimen for a 65 kg patient for treatments administered during a commercial month of 4 weeks (28 days). This study did not consider the disposal after the reconstitution of injectable drugs and cost changes related to the reduction of drug dosages or additional costs resulting from the treatment of adverse events.

### Structural cost management and long-term strategies in the health value chain

**Fig. 3:**
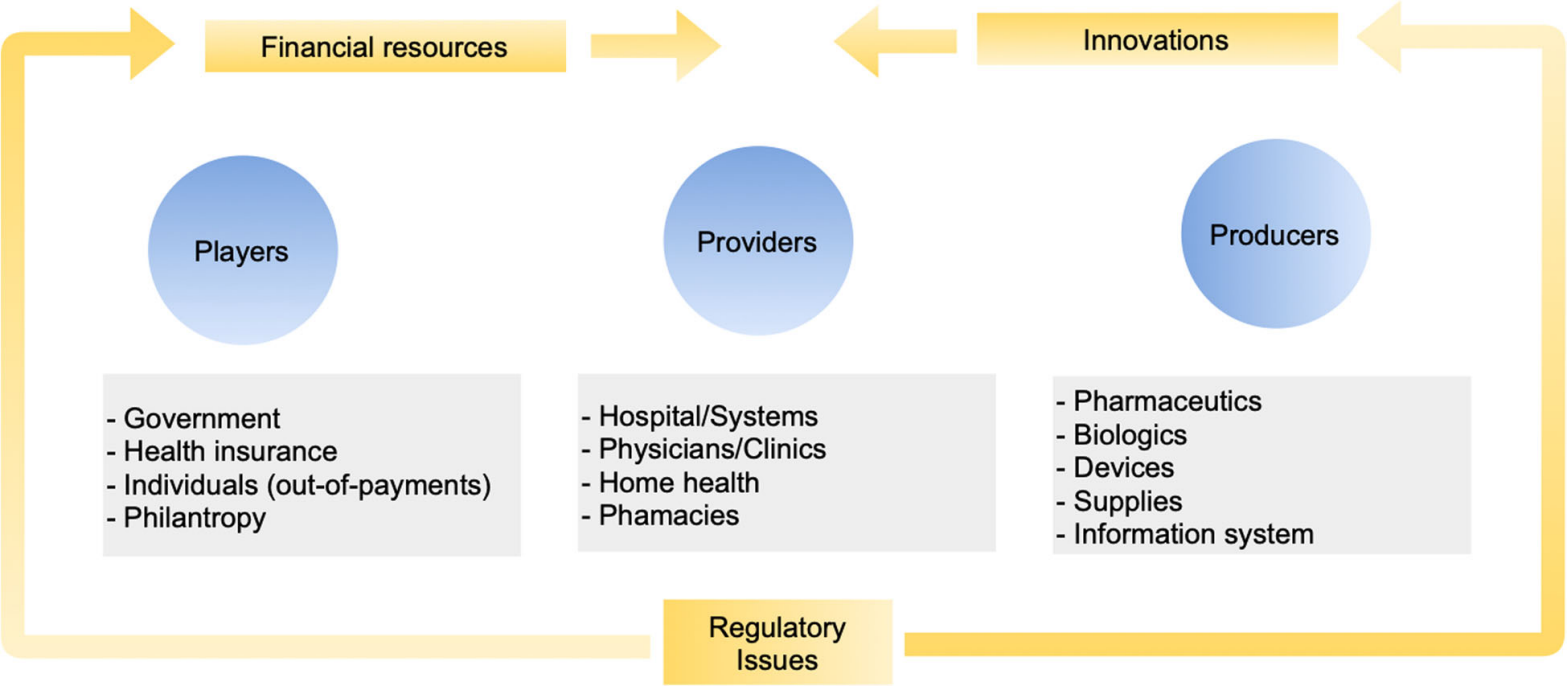
Brazilian healthcare value chain (adapted from Burns, 2018) [48]

We considered the value chain in healthcare with three key sets of stakeholders: individuals and institutions that pay for healthcare, healthcare service providers, and health innovation producers [48]. While the origin of financial resources in the healthcare system is on the left side of Fig. 3, the innovations begin on the far right side. The two flows collide in the middle. In other words, healthcare service providers choose innovation from the right side that they can use in patient treatment given the limited amount of funds received from the left side [48]. As health systems and regulatory approval for access to health innovations in Brazil are issues regulated by the government, we added “regulatory issues” to Fig. 3.

Irrespective of whether it is right or inappropriate care, the type of health care provided is defined by its cost and the budget available to fund it. The Brazilian health system will always attempt to be sustainable; it fits in with its offers [49]. Thus, any change in the health value chain requires a long-term strategy compatible with available cost structures.

Changes in the health value chain from the SCM perspective may involve raising funds from new sources or reallocating existing funds to a certain level where resources are required. Innovations can change the patterns of resource utilization and the cost for HMO patients. Therefore, resource utilization patterns for CM diagnosis and treatment are rapidly changing, and the difference between the SUS and HMO perspective can become more pronounced regarding access to health innovations and medical assistance [21]. Since outputs, not inputs, should measure the health value chain, patients’ health outcomes are what matter, not the volume of services delivered. Comparatively, outcomes and costs are not independent in the healthcare system [50, 51], and the available health resources are limited.

It is plausible to argue successfully that access to appropriate health care is a fundamental human right. In a perfect world, health care would be provided to everyone that needs it when they need it. The reality is that health care, no matter where it is provided, operates in an environment of constraints that requires all stakeholders to rethink long-term strategies regarding the re-engineering of the health value chain. A new value chain has the potential to change the cost structure of the healthcare system and make it more efficient and sustainable. In this sense, in the last part of the study, we used the cost drivers’ results and Brazilian health system publications to evaluate the main barriers to advancing efficiency and cost reduction in the health value chain.

## Results

### Disease model and transition probabilities

**Fig. 4:**
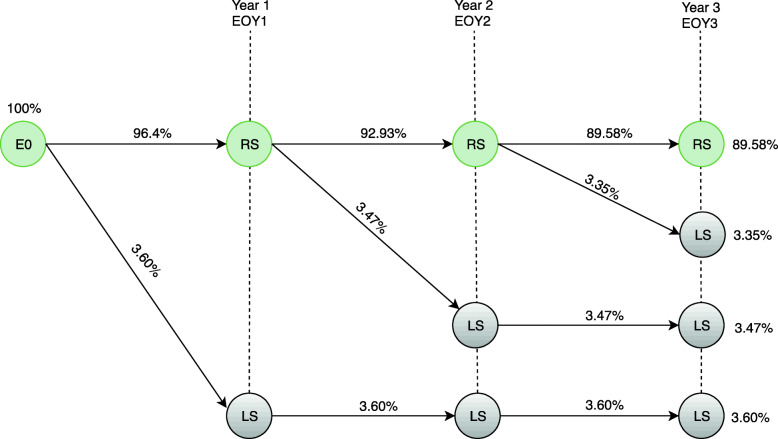
Transition probabilities model for the first 3 years after initial stage 0 diagnosis in the HMO and SUS. E0 = stage 0; RS = pacients remain in the system; LS = patient leave the system; E0Y1 = stage 0 first year; E0Y2 = stage 0 s year; E0Y3 = stage 0 third year

Figure 4 shows the disease model and the general transition probabilities for the first 3 years after initial stage 0 diagnosis from the SUS and HMO perspectives. At this early stage of the disease, recurrence-free survival rates are high. Therefore, the model used only the percentage of all-cause mortality in the Brazilian population over 60 years of age to define the patient’s probability of leaving the system [7, 24], which means death from any cause. Thus, at the end of 3 years, the probability of a CM patient diagnosed with stage 0 remaining in the system is 89.58%, while the probability of the patient leaving the system is 10.42%.

**Fig. 5:**
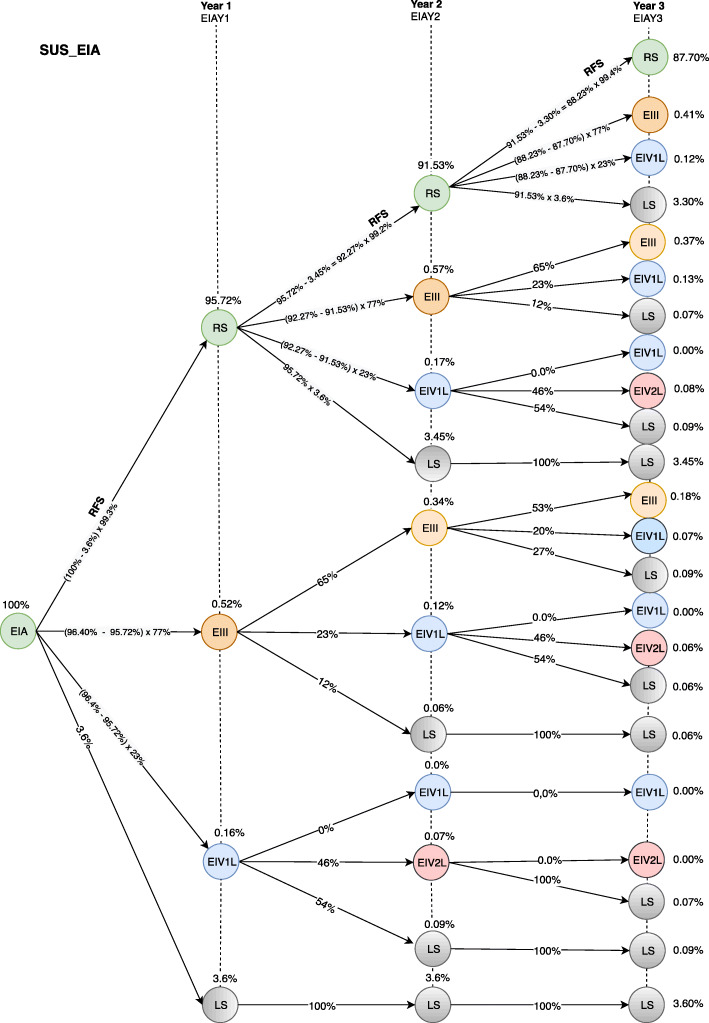
Transition probabilities model for the first 3 years after initial stage IA diagnosis in the public health care system (SUS). EIA = stage IA; RS = pacient remain in the system; EIII = stage rIII; EIV1L = stage unIII+IV first line of treatment (dacarbazine); LS = patient leave the system; EIV2L = stage unIII+IV second line of treatment (palliative care); EIAY1 = stage IA first year; EIAY2 = stage IA second year; EIAY3 = stage IA third year; RFS = relapse free survival

**Fig. 6:**
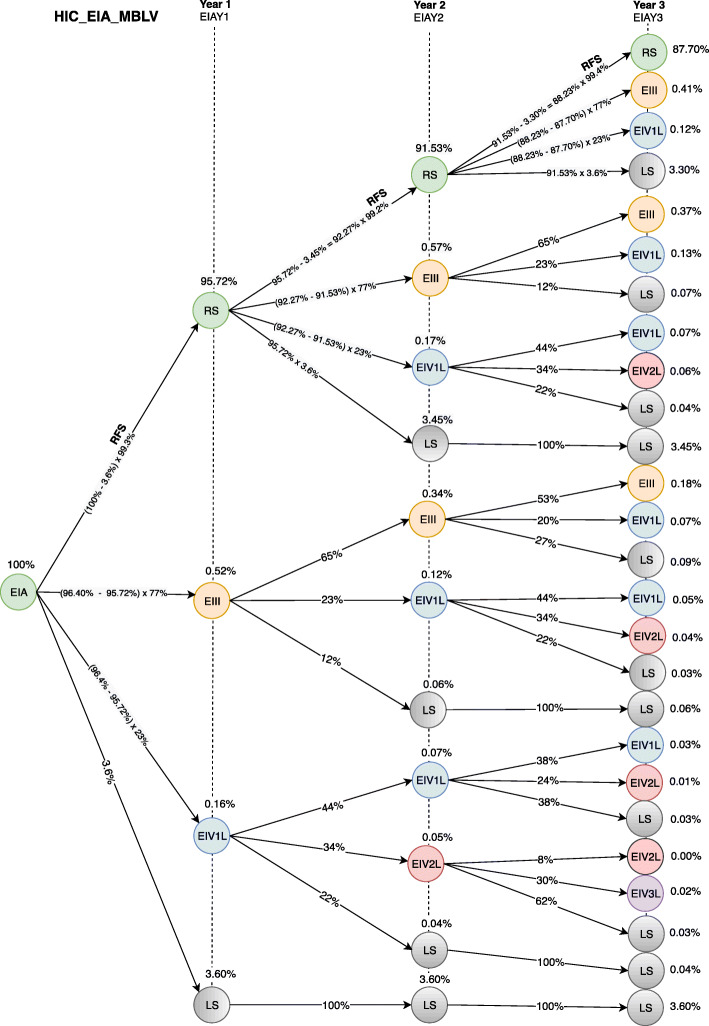
Transition probabilities model for the first 3 years after initial stage IA diagnosis in the private health care system (HMO) to BRAF-mutant patients with low-volume disease (MBLV). EIA = stage IA; RS = pacients remains in the system; EIII = stage rIII; EIV1L = stage unIII+IV first line of treatment (anti-PD-1); LS = patients leaves the system; EIV2L = stage unIII+IV second line of treatment (combined BRAF and MEK inhibition); EIV3L = stage unIII+IV third line of treatment (anti-CTLA-4); EIAY1 = stage IA first year; EIAY2 = stage IA second year; EIAY3 = stage IA third year; RFS = relapse-free survival

Figures 5 and 6 show the transition probability models for the first 3 years after the initial diagnosis at stage IA from the SUS and HMO (MBLV) perspectives. CM patients can be diagnosed at stage IA with the probability of remaining free of recurrence (RS), having recurrence for locally advanced disease (EIII), having recurrence for metastatic disease in the first treatment line (IV1L) or dying from any cause (LS) over a three-yearfollow-up. After migrating to IVL1, the disease model analyzes the probability of recurrence-free survival and overall survival according to the health resource’s performance in the second (IV2L) and third treatment line (IV3L).

According to Figs. 5 and 6, the probability of a CM patient achieving recurrence-free survival in the system after 3 years of stage IA diagnosis is 87.70%, while the probability of transition to stage rIII is 0.96%. The different results between Figs. 5 and 6 regarding the probability of transition at stage unIII + IV (0.46, SUS and 0.60, HMO) are due to different performance of the available resources from the HMO perspective. The diagnostic and therapeutic resources are limited by the underfunding from the SUS perspective. In contrast, HMO oncologists have access to a wider variety of resources that can be used in accordance with the latest scientific advances [21]. The patient’s probability of leaving the system is 10.88 and 10.74% from the SUS and HMO perspective, respectively.

To simulate real life, the model demonstrates that the more advanced the disease stage at the initial diagnosis, the greater the likelihood that the patient will migrate to stages rIII and unIII + IV over the 3 years. Thus, the more advanced the disease, the more significant the transition probability models between the SUS and HMO perspectives will be for the same initial diagnosis stage.

### Executional cost management: financial burden of complete health care delivery cycle

Tables 1 and 2 show the potential financial burden of CM patients at different disease stages from diagnosis up to 3 years of follow up under the SUS and HMO perspectives, respectively. These results confirm previous studies conducted in other countries [52–54] and in Brazil [55] on the considerable rise in costs according to the disease evolution stage at diagnosis. There is only one exception, for the unIII + IV stage in SUS patients whose cost reduction is due to poor performance of available systemic therapy (PFS rate 12 months = 0%). The largest differences by stages were found between 0-IA, in which costs increased 17-fold (SUS) and 12-fold (HMO). Overall, spending on advanced disease patients can be up to 34-fold (SUS) or 270-fold (HMO) higher than that required for the early-stage disease.

**Table 1:** Potential financial burden of CM patients at different disease stages from diagnosis up to 3 years of follow up under the SUS perspective

Stage	Outpatient visit office		Laboratory exam		Imaging exam		Surgery/ pathological analysis		Cancer Drugs		TOTAL
(SUS)	(R$^a^)	%	(R$)	%	(R$)	%	(R$)	%	(R$)	%	(R$)
0	75	21%	0	0%	0	0%	283	79%	0	0%	359 ± 35
IA	116	1.9%	158	2.5%	2574	41.4%	3269	52.6%	99	1.6%	6218 ± 621
IB	119	1.5%	179	3%	3043	37.7%	4245	52.5%	432	5.4%	8022 ± 802
II	129	1.4%	257	2.7%	3147	33.6%	4245	45.4%	1,6	16.9%	9365 ± 936
rIII	134	1.1%	583	4.7%	3145	25.6%	2861	23.3%	5,6	45.3%	12,285 ± 1228
unIII+IV	85	1.1%	419	5.2%	6865	85.1%	701	8.6%	0	0%	8070 ± 807

^a^Brazilian currency: Reais (R$), R$1,0 = USD0,191,898;

Costs estimated by the SIGTAP table according to medical procedures most commonly applied

**Table 2:** Potential financial burden of CM patients at different disease stages from diagnosis up to 3 years of follow up under the HMO perspective

Stage	Outpatient visit office		Laboratory exam		Imaging exam		Surgery/ pathological analysis		Cancer drugs		TOTAL
(HMO)	(R$^a^)	%	(R$)	%	(R$)	%	(R$)	%	(R$)	%	(R$)
0	832	26.0%	0	0.0%	0	0.0%	2321	74%	0	0,0%	3153 ± 514
IA, MBLV	1327	3.5%	723	1.9%	14,515	38.4%	15,557	41%	5797.11	15.2%	37,921 ± 6192
IA, MBHV	1327	3.4%	723	1.9%	14,515	37.6%	15,557	40%	6604.87	17,1%	38,729 ± 6324
IA, WBLV	1327	3.5%	723	1.9%	14,515	38.5%	15,557	42%	5264.82	14,1%	37,389 ± 6105
IA, WBHV	1327	3.4%	724	1.8%	14,515	36.4%	15,557	40%	7220.08	18,4%	39,345 ± 6425
IB, MBLV	1370	1.8%	837	1.1%	20,318	27.0%	26,769	36%	25,327.32	34,1%	74,263 ± 12,185
IB, MBHV	1369	1.8%	834	1.1%	20,271	26.2%	26,769	34.5%	28,164.08	36,4%	77,409 ± 12,640
IB, WBLV	1371	1.9%	838	1.2%	20,324	28.2%	26,769	37%	22,866.88	31,7%	72,169 ± 11,785
IB, WBHV	1370	1.7%	836	1.0%	20,277	25.8%	26,769	33%	30,782.24	38,5%	80,036 ± 13,069
II, MBLV	1797	1.2%	1257	0.8%	28,357	18.2%	26,769	17%	97,858.77	62,8%	156,042 ± 25,481
II, MBHV	1791	1.2%	1245	0.7%	28,189	17.0%	26,769	16%	108,325.88	65,1%	166,322 ± 27,160
II, WBLV	1798	1.2%	1259	0.9%	28,379	19.9%	26,769	18%	87,442.74	60,0%	145,65 ± 23,784
II, WBHV	1796	1.0%	1255	0.7%	28,220	16.3%	26,769	15%	117,899.63	67,0%	175,942 ± 28,731
rIII, MBLV	2868	0.6%	3173	0.7%	64,561	13.2%	17,753	3,6%	399,290.29	81,9%	487,646 ± 28,731
rIII, MBHV	2843	0.5%	3121	0.6%	63,823	12.2%	17,753	3,4%	435,395.04	83,3%	522,937 ± 79,632
rIII, WBLV	2875	0.7%	3188	0.7%	64,715	14.9%	17,753	4,1%	345,670.52	79,6%	434,204 ± 85,395
rIII, WBHV	2873	0.5%	3183	0.6%	64,073	11.5%	17,753	3,2%	469,099.15	84,2%	556,983 ± 70,905
IV*, MBLV	2022	0.3%	4208	0.6%	63,169	8.6%	4797	0.6%	660,997.50	89,9%	735,195 ± 120,056
IV*, MBHV	1951	0.3%	4061	0.6%	60,850	8.5%	4797	0.6%	643,017.51	90,0%	714,677 ± 116,706
IV*, WBLV	2083	0.4%	4332	0.8%	64,476	12.3%	4797	0.90%	460,343.78	85,6%	536,033 ± 87,533
IV*, WBHV	2161	0.3%	4493	0.5%	65,142	7.6%	4797	0.60%	774,093.01	91,0%	850,686 ± 138,916

^a^Brazilian currency: Reais (R$), R$1,0 = USD0,191,898;

IV*: Stage unIII+IV; Costs estimated by the CBHPM table according to medical procedures most commonly applied

Surgery is the main treatment option for most CM patients and usually cures the early-stage disease. The main cost drivers for SUS patients at stages 0, IA, IB, and II were surgery and pathological analysis, accounting for 79, 53, 53, and 45% of total costs, respectively. Cancer drug cost was the main cost driver for SUS patients at stage rIII due to the annual probability of an rIII patient migrating to the metastatic stage considering the disease model and transition probabilities (23, 13%, and 6,9% in year 1, 2 and 3, respectively). Finally, an imaging exam remains an integral component of the staging and surveillance of patients with melanoma, and it represented 85% of the total cost for SUS patients at stage unIII+IV. For all other staging, imaging exams were the second main cost driver for SUS patients.

Under the HMO perspective, surgery and pathological analysis were the main cost drivers at stages 0, IA and IB. Cancer drugs represented more than 60% of the total cost at stages II, rIII, and unIII+IV. The present findings showed that the higher cost at advanced stages is due to high-cost systemic treatment, and different treatments provided to MBLV, MBHV, WBLV, and WBHV patients are the main responsible for different costs within the same staging. For example, the difference in the total cost between the four scenarios at stage unIII+IV based on BRAF status and disease volume can be almost 40%. Health technology assessment has shown that high-cost innovative treatments can be differentiated regarding uncertainty in their clinical and cost effectiveness [56]. However, this information has generally not been considered for decision making in Brazil [21].

Lastly, the results show that HMO patient costs are approximately 10 times more than a SUS patient for the early-stage disease due to cost difference between the same resources used. The total cost difference between the two Brazilian healthcare systems is 90-fold in the metastatic disease due to the incorporation of new technologies that are affordable exclusively in the private healthcare system. Under the HMO perspective, the introduction of CM related innovations has been associated with higher costs and better performance in terms of overall survival and progression-free survival compared with dacarbazine (SUS resource).

## Discussion of results

### Structural cost management and long-term strategies for the health value chain

The results of the TDABC approach showed that total costs were highest for HMO patients and for the advanced disease, mainly because of the difference in health resource cost between SUS and HMO and because of variations in the patient’s clinical course, which depends on the type of health resource available in each health system. Of the various challenges to meet the needs of CM patients, the major one is to restructure the health value chain to ensure the correct resource allocation.

Defining the correct resource allocation remains a challenge with regard to HMO CM patients due to specific issues. First, there is a gap between the rapid pace of technological innovation over the last decade and the researcher’s ability to generate evidence adequate for coverage decisions. For stage unIII + IV, costs at HMOs can vary by up to 40% depending on therapeutic choices based on BRAF status and disease volume. Considering that advanced disease is more costly than the early stages, that cost variation can be a determining factor in the effectiveness of the private health system and shows the potential opportunity to incorporate cost-effectiveness assessment into decision-making.

Second, the health value chain’s correct resource allocation requires knowledge of full care costs, not the portion of costs borne by any one actor [11]. Care for CM patients often requires sustained coordination across multiple specialists and facilities [21], and each one tends to measure what is under their direct control in a particular intervention. The current organizational structure in healthcare delivery makes it difficult to estimate costs correctly. That is one of the most important reasons it is poorly measured or not measured at all. Given the massive amount of resources spent in the HMO, significant efforts must be made to achieve a more systematic approach to compiling cost data for the cost drivers’ knowledge and delivering the right mix of medical care goods and services using available resources. The trend of HMOs to become vertically integrated systems in Brazil, combined with the implementation of functional information systems in the capture of strategic information, will allow managers to enjoy efficient and strategic management of executional costs.

Efficient resource management in the HMO health value chain also involves setting the right time on the patient’s journey to make more significant health investments. According to our insights, the main cost drivers are related to systemic treatment use in the advanced disease. However, the literature is controversial on the effectiveness of primary and secondary prevention efforts. Primary prevention efforts to decrease melanoma incidence through behavior changes are less effective than secondary prevention efforts directed at early detection [57]. Additionally, previous studies have shown that CM screening programs have not resulted in any benefits. On the contrary, screening can generate overdiagnosis of lesions, unnecessary treatment, and the psychosocial consequences of being labeled with a cancer diagnosis [58]. This paper was not intended to analyze primary and secondary prevention efforts costs. However, these insights are of great relevance to long-term public health strategies and should be incorporated into future research. Healthcare costs related to prevention are viewed as discretionary and, therefore, they are best reported separately in cost-of-illness studies [14]. Likewise, future research should consider the cost of drug toxicity, indirect costs, and other direct nonmedical costs at different disease stages to gain a better understanding of the Brazilian private system’s reality.

Finally, efficient resource management in the health value chain of HMO also requires a transparent, ethical and responsible relationship between all stakeholders. While oncology payment mechanisms vary widely across nations depending on their health care systems’ structure, the challenges of appropriate resource selection and patient engagement are common to all [59]. Previous works [59, 60] have shown the advantages of changing the reimbursement model from fee-for-service payment to bundled care payment models. All physician fees, services, facilities, and drugs over the care cycle are included in a maximum cost limit that varies according to disease and the patient’s initial conditions in the new reimbursement models. It is important to note that examining and establishing the maximum cost limit needs to be an ongoing process to avoid the overuse or underuse of required resources [61]. Cancer drug costs accounted for 60–90% of the total cost of stages II-IV. Therefore, it is essential to highlight that commercial interests shape the availability and use of novel therapies to create the most profitable opportunities [10].

On the other hand, as stated by Waitzkin [62], while capitalism is the primary driver of resource overuse, inequality drives resource underuse. The results of the TDABC approach showed that the total costs of diagnosing and treating SUS patients were lower than for the same disease stages from the HMO perspective. Unlike HMOs, the SUS has protocols and guidelines for melanoma, but the choice of the health resource to be used on CM patients is determined by the sector’s underfunding. Most new health technologies are not accessible in the SUS. For example, dacarbazine remains the only treatment for metastatic melanoma endorsed by the SUS, a chemotherapy with clinical results far inferior to new technologies [29, 30]. Thus, in general, the cost of a CM patient is lower in the SUS because of the lower cost of the health resources and/or the limited time that the patient continues to use the resources, as evaluated by the disease model.

To restructure the SUS health value chain and ensure correct resource allocation in the public health system, it is necessary to rethink the available resources in the SUS guidelines and protocols, which impact the Ministry of Health’s budget as a whole, given the universal nature of the SUS. It is important to emphasize that anti-PD1 treatments have been incorporated by the Ministry of Health in Brazil since late 2020, which concluded after analyzing the clinical evidence that the high efficacy and safety of medicines, as well as the unmet medical need for CM patients, would justify the incorporation of the new technology. The new treatments are not yet available due to the need to make administrative decisions, which should occur in the first months of 2021. Such regulatory reforms are necessary to remedy existing shortfalls in the SUS health value chain and take better advantage of the opportunities provided by health innovations. If all the stakeholders could truly tackle the restructuring of the health value chain as their central goal, the resulting improvements in healthcare delivery would break the current stalemate that threatens Brazil’s human and economic health.

Lastly, we are aware that there are many uncertainties in the cost-of-illness studies report for the choice of cost components, quality of data, number of assumptions and methods used to quantify and evaluate costs. While current cost measurement efforts are not perfect given the limitations of this work, the process has begun. It opens up a range of future research options for achieving progress for the SCM literature applied to healthcare.

## Conclusion

CM diagnosis and treatment represent a critical financial burden in Brazil, and the cost of illness varied by stage at diagnosis and between Brazilian health care systems. The cost-of-illness study for CM has the potential to inform policymakers and decision-makers regarding the economic burden that melanoma imposes on a society in terms of use of health care services, assisting them in making projections of future health care costs and in resource allocation decisions. We believe that cost-of-illness analysis from a strategic perspective may also aid the assessment of executional costs and can be used to support the change in structural costs required for long-term strategies related to the health value chain.

## Supplementary Information

**Additional file 1.**

**Additional file 2.**

## Data Availability

The datasets supporting this article’s conclusions and more information on the differences between the SUS and HMO are included in Supplementary Material 1. Supplementary Material 2 includes the checklist for conducting cost-of-illness studies.

## Abbreviations

CM: Cutaneous melanoma
SUS: Unified Health System
SCM: Strategic Cost Management
HMO: Health Management Organization
TDABC: Time-Driven Activity-Based Costing
APAC: Authorization for High Complexity Procedures
CMED: Ministers of the Drug Market Regulation Chamber
E0: Stage 0
RS: Pacient remains in the system
LS: Patient leaves the system
E0Y1: Stage 0 first year
E0Y2: Stage 0 s year
E0Y3: Stage 0 third year
EIA: Stage IA
EIII: Stage rIII
EIV1L: Stage unIII+IV first line of treatment (dacarbazine)
EIV2L: Stage unIII+IV second line of treatment (palliative care)
EIAY1: Stage IA first year
EIAY2: Stage IA second year
EIAY3: Stage IA third year
RFS: Recurrence-free survival
EIV1L: Stage unIII+IV first line of treatment (anti-PD-1)
EIV2L: Stage unIII+IV second line of treatment (combined BRAF and MEK inhibition)
EIV3L: Stage unIII+IV third line of treatment (anti-CTLA-4)

## Footnotes

1 Operational cost unit (OCU) = R$20.47^38^ Square meter of the film used in radiology and in diagnostic imaging = R$27.02^39^

2 ICMS = Brazilian tax on the circulation of goods, interstate and intercity transportation and communication services.
