# Supplementary material for: Cutaneous melanoma: cost of illness under Brazilian health system perspectives
Source: BMC Health Serv Res. 2021 Mar 29;21:284. doi: 10.1186/s12913-021-06246-1 (PMC8008665; doi:10.1186/s12913-021-06246-1)
Supplement: Supplementary file 2 — Additional file 2. [file 12913_2021_6246_MOESM2_ESM.docx]

**Supplemental material 2**

Cost-of-illness evaluation checklist (based on Larg and Moss, 2011)

**(1) Analytical framework: what costs should have been measured?**

**(a) What was the motivation and perspective of the study?**

The goal of this cost-of-illness study was to analyze and correlate the healthcare resources used for Cutaneous Melanoma (CM) diagnosis and treatment with: (i) short-term tactics through cost driver analysis (executional cost management), and (ii) long-term strategy through the re-engineering of the value chain that was compatible with different cost structures (structural cost management).

The cost-of-illness study for melanoma was conducted from the perspective of two health systems in Brazil: the public health system (Unified Health System, SUS) and the private health system (Health Management Organization, HMO).

**(b) Was the appropriate epidemiological approach taken?**

The cost-of-illness study for melanoma considered the direct medical cost in a bottom-up analysis, which consisted of estimating the types of health resources consumed by an individual throughout their journey with the disease, multiplied by the respective unit costs. The disease’s financial burden was based on the melanoma incidence, knowledge of the disease’s progress and the overall survival rates. The epidemiological approach that was chosen was appropriate because incidence-based studies can show how costs vary according to the duration of the disease, which may be useful when planning interventions targeted at specific stages [14].

**(c) Was the study question well specified?**

**(i) Were all relevant, non-trivial cost components and their stakeholders identified?**

The main cost drivers and the total cost structure of the complete healthcare delivery cycle for CM patients were investigated considering different disease stages and possible variations in their clinical course (based on the disease model). In addition to the total financial burden, the costs were also analyzed based on five groups of different medical resources: outpatient visits, laboratory exams, imaging exams, surgery and histopathological analysis, and drugs.

The unit cost of each resource was obtained through a unified system of information management of procedures, medications, orthoses, prostheses, and special materials (SIGTAP) for the SUS and through the Brazilian hierarchy classification of medical procedures (CBHPM) for HMO. CBHPM allows up to 20% of the procedure cost, according to regionalization and free negotiation between the parties. SIGTAP allows a total percentage of the procedure cost linked to a specific health care provider qualification (more than 10%). To simulate this cost variation, we used Oracle Crystal Ball Software.

The medical procedure codes most commonly applied to the CM patient considering all the pricing rules of the SIGTAP and CHBPM were chosen. Regarding cancer therapy costs, the HMO costs were estimated based on the drug list (ICMS tax 18%) published by Ministers of the Drug Market Regulation Chamber (CMED). Cancer therapy costs in the SUS are controlled by the Authorization for High Complexity Procedures (APAC) system, and they integrate specific policies of the Ministry of Health. Each APAC has a particular sum for reimbursement, according to the type of cancer therapy used and treatment line. When applicable, this study considered a dose regimen for a 65 Kg patient for treatments performed during a commercial month of 4 weeks (28 days). This study did not consider the disposal after reconstitution of injectable drugs and cost changes related to the reduction of drug dosages or the additional cost resulting from treatment for adverse events. Direct non-medical and indirect costs were not included in the study because it is not possible to quantify them by clinical trial data and because of the absence of standardized and reliable data in Brazil.

**(ii) Were necessary timeframes specified?**

We evaluated the financial burden of CM patients at different disease stages from diagnosis up to three years of follow-up from the perspective of two Brazilian payers: SUS and HMO. The disease’s financial burden was based on melanoma incidence, knowledge of the disease’s progress and the overall survival rates from pivotal studies.

**(iii) Was a case of disease or risk factor adequately and appropriately defined?**

In the absence of a national database with a historical series of health resource consumption per patient in Brazil, we used secondary data supported by melanoma literature to propose a disease model inspired by Markov models to compare the disease burden in the different melanoma stages. This division of the melanoma burden may be helpful when determining priorities for healthcare resource allocations.

New CM patients could start at one of the six diagnosis stages, 0, IA, IB, II, rIII (resectable disease) or unIII + IV (stage III unresectable + metastatic disease) in the disease model. They could either stay at the same stage or change to another stage depending on the disease model and transition probabilities. We analyzed the first three years following the diagnosis that was considered when patients were aged 60 years. They could remain in the model until death by malignant tumor or achieve life expectancy.

**(iv) Was the counterfactual population occurrence plausible and meaningful?**

The disease model allowed us to evaluate the costs against a counterfactual scenario in which the new CM patient had a hypothetical alternative occurrence of the health problem that was biologically plausible and realistic based on the natural history of CM, considering the health resource used and the Health system in which he was served. The disease model included the ‘theoretical minimum risk’ distribution, which is the distribution of the health problem that would produce the minimum feasible population disease burden, derived from secondary data supported by the melanoma literature. The assumptions of the time-driven activity-based costing (TDABC) approach ensured that only costs related to CM were attributed to the total cost.

**(2) Methodology and data: how well were resource use and productivity losses measured?**

The assessment of lost productivity is not applicable to the perspective used in the study.

**(a) Were appropriate quantification methods used, so that**

**(i) additional, or excess, costs were measured?**

**(ii) only costs specific to (caused by) the health problem were included (confounders controlled)?**

**(iii) all important effects were captured?**

**(iv) important differences across subpopulations were accounted for?**

**(v) the required level of detail could be provided?**

The measurement of costs considered:

a) The natural history of the disease by stage in the diagnosis: evaluated by the Disease Model based on secondary data supported by the melanoma literature.

b) Process mapping and micro-costing of all health resources used in the diagnosis and treatment of CM patient up to 3 years after diagnosis (executional cost).

The Person-Based Method (‘Bottom-Up’) relies on the availability of data on disease status, as well as current resource use for each individual over the time period of the study. The methodology used presents the best specificity and sensitivity to estimate costs according to the secondary data available for analysis.

**(b) Was the resource quantification method(s) well executed?**

**(i) For population-based studies, were the cost allocation methods, data and assumptions valid?**

**(ii) For person-based studies, were appropriate statistical tests performed and reported?**

**(iii) Were the data representative of the study population?**

**(iv) Were there any other relevant resource quantification issues?**

In the absence of a national database with a historical series of health resource consumption per patient in Brazil, we used a published database with person-level healthcare utilization data for people served by HMO or SUS. Thus, the type and frequency of healthcare resources used in CM patients were evaluated in a previous Brazilian study, which is the only behavioral study applied to a Brazilian group of medical professionals who diagnose and treat melanoma. That study assessed the type and quantity of health resources used for the complete delivery cycle of health care at different stages of CM patient evolution in the Brazilian public and private health system. The Time-Driven Activity Based Cost approach applied to the database ensured that only costs related to the CM were attributed to the total cost. Despite the limitations inherent to using primary data from previous research, the required strategic information would not be accessible otherwise. The study is not population-based. There were no relevant resource quantification issues, but some assumptions were required for the analysis.

**(c) Were healthcare resources valued appropriately?**

Brazilian health care is fragmented by facility or specialty, and this obstacle hinders accurate measurements of costs. To overcome these challenges, we applied the time-driven activity-based costing (TDABC) approach, a feasible tool for comparing relative resource utilization that exploits time equations without increasing the model’s complexity. Another benefit of implementing a TDABC approach is the knowledge it generates on resource utilization efficiencies employing process mapping. The TDABC addresses many executional cost management issues, allowing us to investigate the main cost drivers and the total cost for the complete healthcare delivery cycle using the resource’s consumption time based on the disease model.

The type and frequency of healthcare resources used in CM patients were evaluated in a previous Brazilian study and the resource’s consumption time was based on the disease model. The unit cost of each resource was obtained by a unified system of information management of procedures, medications, orthoses, prostheses, and special materials (SIGTAP) for the SUS and by the Brazilian hierarchy classification of medical procedures (CBHPM) for HMO. CBHPM allows up to 20% of the procedure’s cost, according to regionalization and free negotiation between the parties. SIGTAP allows a total percentage of the procedure cost linked to a specific health care provider qualification (more than 10%). To simulate this cost variation, we used Oracle Crystal Ball Software.

The medical procedure codes most commonly applied to the CM patient considering all the pricing rules of the SIGTAP and CHBPM were chosen. Regarding cancer therapy costs, the HMO costs were estimated based on the drug list (ICMS tax 18%) published by Ministers of the Drug Market Regulation Chamber (CMED). Cancer therapy costs in the SUS are controlled by the Authorization for High Complexity Procedures (APAC) system, integrating specific policies of the Ministry of Health. Each APAC has a certain sum for reimbursement, according to the type of cancer therapy used and treatment line. When applicable, this study considered a dose regimen for a 65 Kg patient for treatments performed during a commercial month of 4 weeks (28 days). This study did not consider the disposal after reconstitution of injectable drugs and cost changes related to the reduction of drug dosages or the additional cost resulting from the treatment of adverse events. Direct non-medical and indirect costs were not included in the study due because it is not possible to quantify them by clinical trial data and because of the absence of standardized and reliable data in Brazil.

**(d) Was the approach for valuing production losses justified, and the assumptions valid?**

The assessment of lost productivity is not applicable to the perspective used in the study.

**(e) Was the inclusion of intangible costs appropriate:**

**(i) Was the double counting of mortality-related production losses avoided?**

**(ii) Were losses valued appropriately, given the study’s perspective?**

The assessment of intangible costs is not applicable to the perspective used in the study.

**(3) Analysis and reporting**

**(a) Did the analysis address the study question?**

**(b) Was a range of estimates presented?**

**(c) Were the main uncertainties identified?**

**(d) Was a sensitivity analysis performed on:**

**(i) important (uncertain) parameter estimates?**

**(ii) key assumptions? (including the counterfactual)**

**(iii) point estimates? (based on confidence or credible intervals)**

**(e) Was adequate documentation and justification given for cost components, data and sources, assumptions and methods?**

**(f) Was uncertainty around the estimates and its implications adequately discussed?**

**(g) Were important limitations discussed regarding the cost components, data, assumptions and methods?**

**(h)Were the results presented at the appropriate level of detail to answer the study question (cost components; disease subtypes, severity, stage; subpopulation groups, cost bearers)?**

Cost-of-illness studies attempt to quantify the magnitude of an association between disease and cost. We are aware that there are many uncertainties in the cost-of-illness studies report for the choice of cost components, the quality of data, number of assumptions and methods used to quantify and value costs. In general, the study meets the requests of the cost-of-illness evaluation checklist [14]:

1. The results respond to the objective of the study and are presented with an appropriate level of detail to answer the study question;

2. The range of estimates of the secondary data used in the study was presented (supplementary material 1);

3. The main uncertainties, limitations and key assumptions were discussed and are detailed in the study.

4. On the other hand, we did not conduct a sensitivity analysis in the study.

Disease Model assumptions:

Although the model aimed to simulate reality as much as possible, some simplifying assumptions had to be made: (1) transition probabilities were assumed to be the same for both genders and all ages over 60 years; (2) hazard rates for a second cutaneous melanoma were not considered, although previous studies have shown that CM patients may have increased risk of subsequent CM or non-CM; (3) at the time of this study, the most recent available data did not allow the subclassification of stages II and III, but new subcategorization of the staging of melanoma will continue to evolve to enable better care; (4) the diagnosis of CM was made within one month for both HMO and SUS patients, but this time may be longer.

TDABC Approach assumptions:

When applicable, the TDABC approach considered a dose regimen for a 65 kg patient for treatments performed during a commercial month of 4 weeks (28 days). This study did not consider the disposal after reconstitution of injectable drugs and cost changes related to the reduction of drug dosages or the additional cost resulting from the treatment of adverse events.
